# Observation of the Breeding Behavior of the Chinese Giant Salamander (*Andrias davidianus*) Using a Digital Monitoring System

**DOI:** 10.3390/ani8100161

**Published:** 2018-09-25

**Authors:** Qinghua Luo, Fang Tong, Yingjie Song, Han Wang, Maolin Du, Hongbing Ji

**Affiliations:** 1 Hunan Engineering Laboratory for Chinese Giant Salamander’s Resource Protection and Comprehensive Utilization, Jishou University, Zhangjiajie 427000, China; Tongfang20180426@126.com (F.T.); yjsong0517@163.com (Y.S.); wanghan20180426@126.com (H.W.); 2 School of Energy and Environmental Engineering, University of Science and Technology Beijing, Beijing 100083, China; jih_0000@126.com; 3 Key Laboratory of Hunan Forest Products and Chemical Industry Engineering, Jishou University, Zhangjiajie 427000, China; 4 Zhangjiajie Zhuyuan Biological Technology of Chinese Giant Salamander Co. Ltd., Zhangjiajie 427000, China; sttf0024@126.com

**Keywords:** breeding behavior, sand-pushing, courtship, oviposition, parental care, showering, Chinese giant salamander (*Andrias davidianus*)

## Abstract

**Simple Summary:**

Behavioral research on wild Chinese giant salamanders (*Andrias davidianus)* is in its infancy because *A. davidianus* inhabit underground river dens that are difficult to access. In order to ascertain the types of reproductive behavior exhibited by *A. davidianus*, this paper monitored their reproductive activity using a digital monitoring system in a simulated natural habitat. The survey uncovered reproductive behavior such as sand-pushing, showering, courtship, oviposition, and parental care. We also recorded the parental care time allocation for the first time. This study provides a scientific basis for the method optimization for the ecological reproduction of *A. davidianus* and the conservation of its wild population. This study also demonstrates that a digital monitoring system is an effective research method for investigating the behavior of Cryptobranchidae and other cave animals.

**Abstract:**

Knowledge of natural animal behavior is essential for enhancing the protection and artificial breeding of animals. At present, the behavior of the Chinese giant salamander (*Andrias davidianus*) is studied through interviews with local people or occasional observations under artificial conditions, leading to a lack of systematic records. Thus, most reports are descriptive and lack quantitative analyses. To ascertain the types of reproductive activities and their corresponding time allocations, this study observed the reproductive behavior of *A. davidianus* using a digital monitoring system for the first time. The results showed that sand-pushing behavior is mainly carried out by the limbs, tail, head, and body of den-dominant males. Showering behaviors included rinsing the trunk, head, and tail. Courtship was composed of a series of behaviors, including standing side-by-side, belly colliding, mounting, mouth-to-mouth posturing, chasing, inviting, cohabitating, and rolling over. After chasing and interlocking with the male, the female discharged her eggs. The oviposition process began at either 02:04 or 04:09, and lasted either 66 or 182 min. Parental care included tail fanning, agitation, shaking, and eating dead and unfertilized eggs, and the durations of these behaviors accounted for 31.74 ± 4.35%, 17.42 ± 4.00%, 3.85 ± 1.18%, and 1.19 ± 0.69% of the entire incubation period, respectively. This paper reveals the characteristics of the reproductive behavior of *A. davidianus* and provides a scientific basis for the management of its ecological breeding and the conservation of its wild populations.

## 1. Introduction

Cryptobranchids (giant salamanders) live in cool flowing water and use external fertilization. The family consists of two extant genera and three currently recognized species. The Chinese giant salamander (*Andrias davidianus*) is the largest amphibian in the world, with a maximum length of 1.7 m and a maximum weight of 60 kg [1]. It first appeared in fossil records during the middle Jurassic [2] or early Cretaceous period [3], thus it is an ancient species [4]. Due to threats from over-harvesting and habitat destruction, *A. davidianus* is listed as critically endangered in the IUCN (International Union for the Conservation of Nature) Red List of Threatened Species, and has been protected as a grade two protected species in China since 1988 [5,6].

The Japanese giant salamander (*Andrias japonicus*) is a closely related species and is slightly smaller than *A. davidianus*. The North American giant salamander (Hellbender; *Cryptobranchus alleganiensis*) reaches only ~30% of the total length, and ~9% of the weight of the *Andrias* spp. [7]. Cryptobranchids are similar in their biology, including extreme longevity, highly conserved morphology, low metabolism, parental care by males, and large larvae [8]. However, there are differences in their habitats, diets, reproductive behavior, seasonality, fecundity, egg size, and mating strategies [8]. *A. davidianus* spawn in dens that are large hollows beneath rocks or in riverbanks, and usually have an entrance [9]. In contrast, *C. alleganiensis* spawn in hollow dens under rocks and in crevices. Moreover, the spawning dens of *A. japonicus* are often found in very small streams at the upper reaches of tributaries [10,11].

Although some papers have been published on the reproductive behavior of *A. davidianus*, the literature is scattered, with many important articles only written in the national languages of the bio-political regions where the species are found, except for a recent review in Browne et al. [8]. Previous research on the reproductive behavior of *A. davidianus* was conducted through surveys or occasional observations [12,13,14,15,16,17]. Male and female *A. davidianus* demonstrated behaviors that included mouth-to-mouth posturing (touching each other with their snouts), gore-abdomen (the male pushes the belly of the female), sporting (playing together), and pairing (mating), after they were injected with sex hormones during breeding season [15]. In simulated natural habitats, the reproductive behavior includes sand-pushing (the male pushes sand out of the den), courtship (choosing the partner), showering (males rinse their body with flowing water), mating, and egg-safeguarding (males safeguard eggs during the hatching process) [16]. Mating behavior is comprised of females entering the spawning den, head exposing, inspection, trailing, cloacal scenting, mouth contact, and males riding the back of females [16]. There was a positive correlation between showering and courtship behaviors [17].

Wild populations of *A. davidianus* inhabit dens in riverbanks or underground rivers, which are very difficult to access [18]. Although a large number of farms have been breeding salamanders for the past few decades [6], the level of breeding methodologies is suboptimal, the reproductive rate is low and unstable, and each parental *A. davidianus* only reproduces 64.9 ± 52.6 hatchlings [19]. Therefore, their breeding methodologies must be improved. In this paper, the reproductive behavior of the *A. davidianus* was monitored with an infrared surveillance system in a simulated natural habitat. We aimed to ascertain the types and routines of reproductive behavior, which could provide a basis for the optimization of reproductive methods and the conservation of wild populations.

## 2. Materials and Methods

### 2.1. Study Site

We chose two artificial streams at a simulated ecology farm in Zhangjiajie, Hunan Province (29°28′ N, 110°22′ E, 480 m a.s.l.), to observe the reproductive behaviors of *A. davidianus*. The length and width of each stream were 8 m and 1.1 m respectively, with sand, pebbles and some soil on the bottom. Five dens were built on each side of the streams, with plants growing on the top and sand and pebbles on the bottom. Each den was cuboid-shaped and was 1.2 m in length and width, and 0.5 m in height, with an entrance of 0.25 m in height, 0.3 m in width and 0.2 m in length. The water depths were 0.4 m in the stream and 0.3 m in the dens. There were eight salamanders (aged 8–9, weighing between 6–8 kg) in each stream, with a 1:1 sex ratio (See Table A1 in Appendix A for their characteristics).

### 2.2. Digital Monitoring System

We randomly selected three dens from each simulated stream, and placed infrared video cameras (HIKVISION DS-2CD3T35D-I5, effective pixels 2048 × 1536, 4 mm lens, Hangzhou Hikivision Digital Technology Co. Ltd., Hangzhou, China) at the top of them by opening their removable lids. In addition, one camera was installed above each stream to observe behavior outside of the dens. Past records show that *A. davidianus* lay eggs during late August at this farm. Therefore, we set up 24 h recordings from 27 July to 1 October 2016. Altogether, there were recordings from eight cameras for 67 days in 2016.

### 2.3. Observation Index

**Figure 1:**
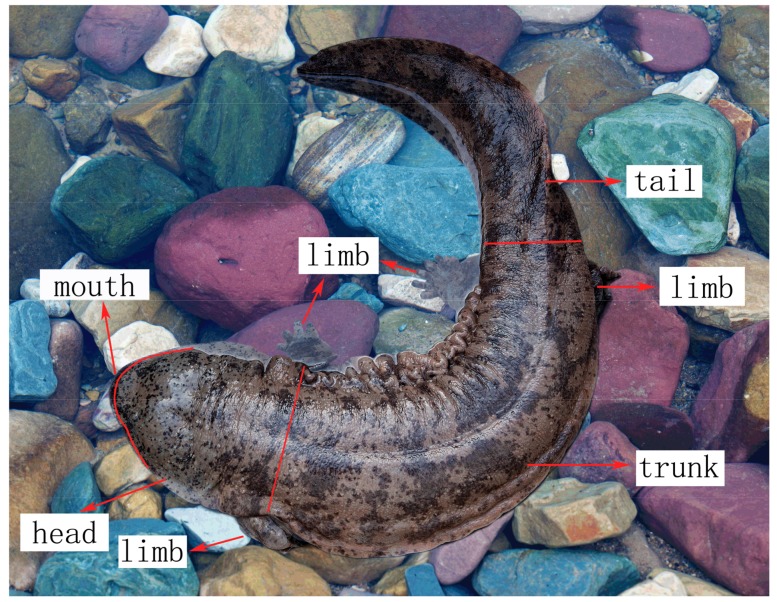
Body parts of *Andrias davidianus*.

All behavior that occurred during the observation period were record to quantify the type of behavior, duration of continuous activities, and frequency of each behavior. A series of states and events were recorded separately, and then combined to detect patterns. All behavioral frequencies were recorded for males and females. To categorize the location of the event type, the body was divided into five regions: mouth, head, trunk, limbs, and tail (Figure 1).

Reproductive behavior characteristics of *A. davidianus* were systematically monitored. The detailed nocturnal activity rhythms will be reported in another manuscript. The characteristics of reproductive behaviors including sand-pushing, showering, courtship, oviposition, and parental care, and the time allocation of each kind of parental care behavior were studied in detail.

### 2.4. Data Processing

The data were statistically analyzed using the SPSS 20.0 package. The incubation time was divided equally into early, middle and late stages. The interval sampling of time allocation for each parental care behavior was recorded for each stage. A one-way ANOVA was performed to study the differences in the time allocation of each parental care behavior between different stages.

## 3. Results

### 3.1. Sand-Pushing

**Figure 2:**
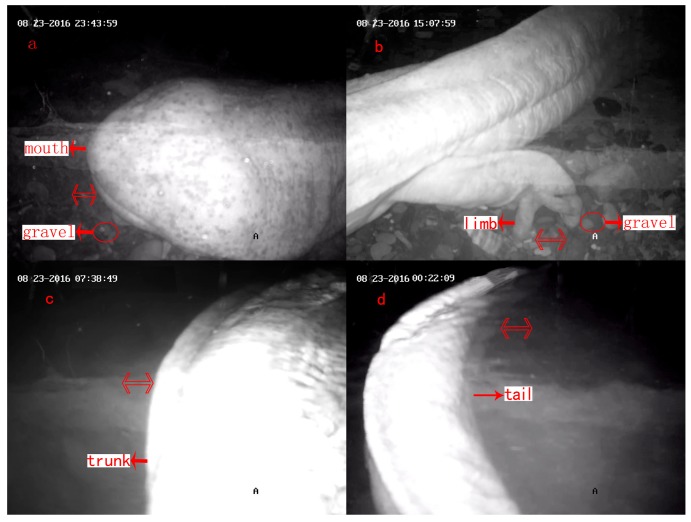
Sand-pushing behavior: gravel was pushed out of the den or to the corner of the den using the mouth (**a**), limbs (**b**), trunk (**c**), and/or tail (**d**). Note: Photos are screenshots of the monitoring video. The two-way arrows on the photos represent the moving direction. This is also true for Figure 3, Figure 5 and Figure 6.

One male entered the den and began to push sand out of the den from 22 August 2016. He pushed sand for nearly an entire day on 23 August, and the shape of the den changed on 24 August. This pushing behavior finished on 30 August, and lasted for eight days. After the sand-pushing behavior, the bottom of the den was clean, smooth and depressed, which allowed the body of the male to be submerged in the water. This male could be a successful den master, attracting females to come and lay eggs. The male pushed gravel with his head, limbs, trunk, and tail, modifying his den (Table 1, Figure 2), which took 4.2 (6.2%), 36.3 (53.8%), 2.1 (3.1%), and 24.9 (36.9%) hours, respectively.

### 3.2. Showering Behavior

**Figure 3:**
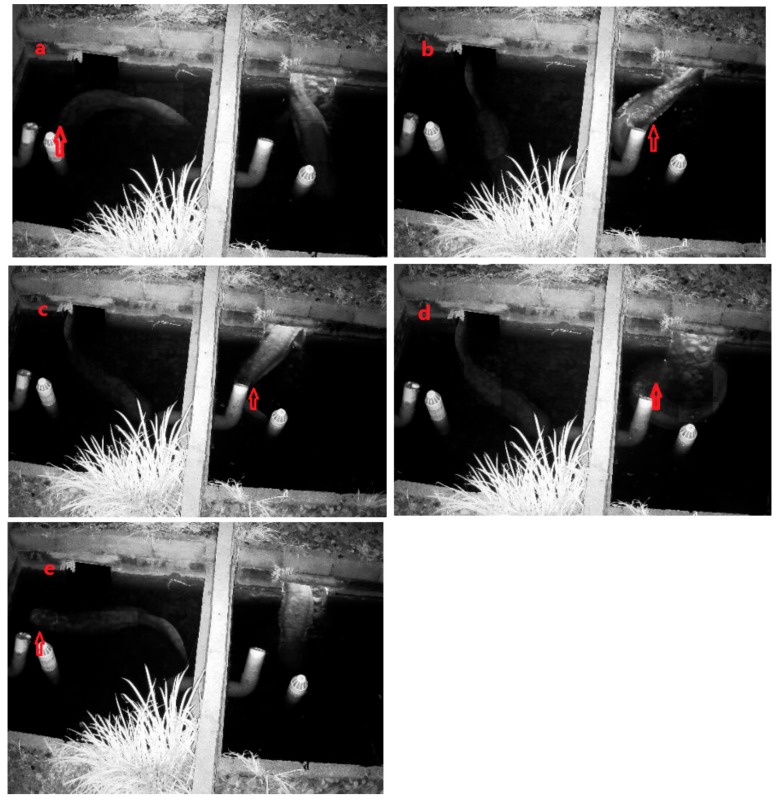
Showering behavior of a male *A. davidianus*: (**a**) head; (**b**) trunk; (**c**) tail; (**d**) curve; and (**e**) snout.

**Figure 4:**
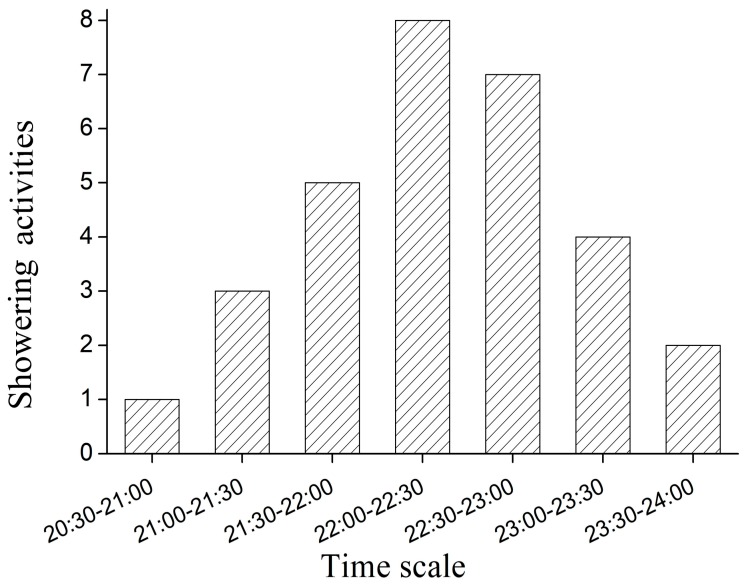
Times of showering behavior of male *A. davidianus*.

Showering behavior was defined as males leaving the den to rinse their head, trunk and tail under the flow of water from the outlet of a water pipe. Thirty self-rinsing activities were observed in two males from 27 July to 26 August 2016. The actions involved rinsing the head, trunk and/or tail (Table 2, Figure 3). The order, duration and proportion of each showering behavior was as follows: trunk > tail > head > curve> snout. The showering behavior occurred from 20:30 to 24:00, with peak showering behavior occurring between 22:00–23:00 (Figure 4).

### 3.3. Courtship Behavior

Courtship behavior began one month before laying eggs, between 20:00 and 23:00, with a peak time of 21:30–22:30. Courtship behaviors included knocking bellies, leaning side-by-side, riding, mouth-to-mouth posturing, chasing, rolling over, inviting, and cohabiting (Table 3). The courtship activities of two pairs of *A. davidianus* were observed. The order of each behavior, by frequency, was as follows: leaning side-by-side > knocking bellies > riding > mouth-to-mouth posturing > chasing > inviting > cohabiting > rolling over. The duration percentages of the eight courtship behaviors were as follows: cohabiting > leaning side-by-side > knocking bellies > riding > mouth-to-mouth posturing > inviting > chasing > rolling over (Table 3). Cohabiting lasted the longest at 88.32%, followed by standing side-by-side at 2.83%. Among the duration of each continuous behavior, cohabiting lasted the longest at 181.25 ± 38.67 min, followed by chasing at 3.04 ± 0.57 min. Mouth-to-mouth posturing lasted the shortest time, at 1.60 ± 0.24 min (Table 3).

### 3.4. Oviposition

**Figure 5:**
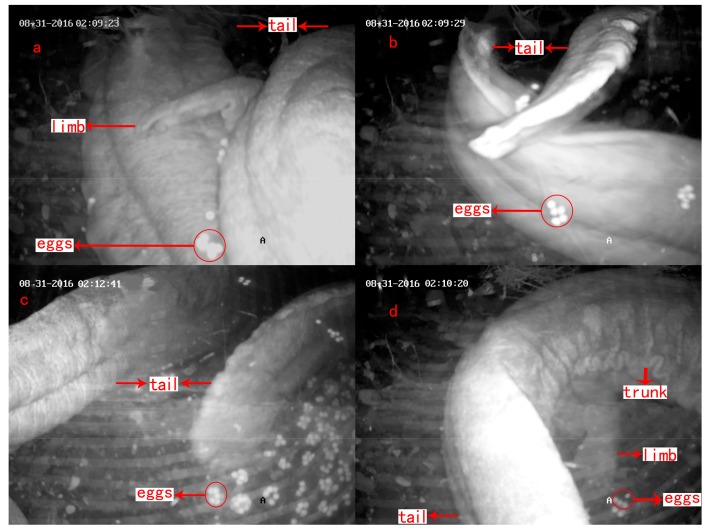
Process of oviposition in *A. davidianus*: (**a**) the female begins discharging eggs; (**b**) the male and the female’s tails cross; (**c**) the male moves to the entrance of the den; and (**d**) the female body bends into a U-shape.

Male A exited and entered the den four times between 01:00–01:50 on 23 August 2016, and male B exited and entered his den six times between 03:00–03:50 on 31 August 2016. Male A’s partner, female A, entered the den at 02:04, and female B entered male B’s den at 04:09. Then, each pair intertwined and chased each other in the den for 7 min and 5 min, respectively. After resting for approximately 0.58 min and 0.41 min, respectively, the males strongly beat the females’ sides with their heads, then positioned themselves under the bellies of the females and lifted them up. The belly of female A began to shake and tremble, then she discharged eggs (Figure 5a) for the first time at 02:09. Female B discharged her eggs at 04:25 while her male partner touched her belly with his head and their tails crossed (Figure 5b). This process lasted for 1–3 min before the male ejaculated semen and the water quickly became turbid. After the female rested for 25–150 s, oviposition and spermiation occurred for a second time and then continued. In pair A, oviposition and spermiation occurred twice. In pair B, this occurred seven times. Later, the male moved to the entrance of the den (Figure 5c), while the female rotated in the den, with her body bent into a U-shape (Figure 5d). The entire oviposition process of pair A lasted for 66 min, from 02:04 to 03:10, and pair B lasted 182 min, from 04:09 to 07:11. The female left after oviposition, and the male stayed in the den to care for the eggs.

### 3.5. Parental Care

**Figure 6:**
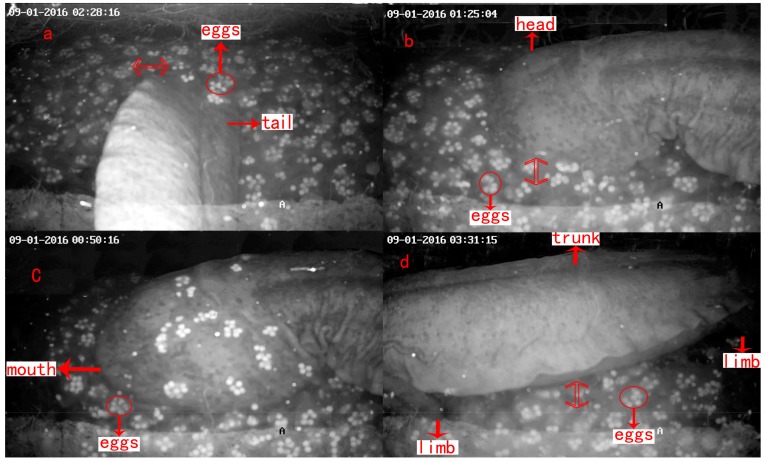
Parental care behavior in male *A. davidianus*: (**a**) tail fanning; (**b**) agitation; (**c**) egg eating; and (**d**) shaking.

With a daily mean WT (water temperature) of 18.41 ± 1.99 °C and 18.72 ± 1.91 °C, the incubation process lasted 45–50 days (from 16 August to 1–4 October and 31 August to 15–16 October). The den master defended the nest and guaranteed the hatching of the eggs using tail fanning, agitation, shaking, and egg eating, with each behavior lasting 2.1–3.5 min (Table 4 and Figure 6). The males also stayed still, opened and closed their mouths for no obvious reason, and moved in and out of the den.

**Figure 7:**
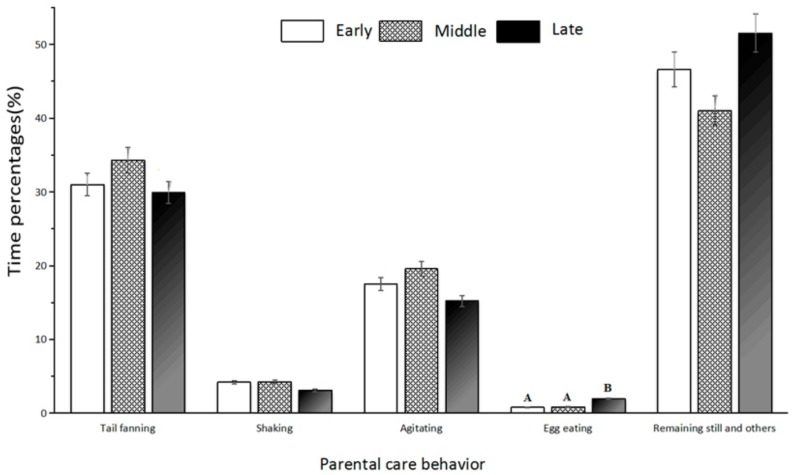
Time percentages of parental care behavior in *A. davidianus.* Note: The different codes after the average number in the same column indicate that there are differences among groups. The capital letters represent *p <* 0.01.

Five days were selected to record the time distribution for each behavior of parental care, which were from the early, middle and late stages, with an interval of two days. During all three stages, the durations of the different parental care behaviors were ranked as follows: tail fanning (31.74 ± 4.35%) > agitation (17.42 ± 4.00%) > shaking (3.85 ± 1.18%) > egg eating (1.19 ± 0.69%). Tail fanning was more frequent than the other behaviors. The results from a one-way ANOVA showed that there was only one significant difference (*p* < 0.01) in the time distribution of egg eating among the early/middle and late stages (Figure 7).

## 4. Discussion

### 4.1. Sand-Pushing

Our observations confirm previous descriptions by Liu (1990) [12] and Liang et al. (2010) [16] which describe the male *A. davidianus* cleaning gravel and sand from the den with their heads and tails prior to female entrance. We found that the behavior was mainly implemented by the limbs and tail rather than the head and body, due to the total duration and stronger digging force provided by the limbs and tail. In addition, different types of sand-pushing behavior possibly moved different sizes of gravel out of the den and from different locations. Sand-pushing modifies the bottom of the den into a larger space and increases the depth of the water inside the den, which may be essential for oviposition and to keep more eggs submerged in water [16,17]. Moreover, it removes mud and provides a clean environment for egg incubation, possibly enhancing the incubation rate [16,17]. Sand-pushing may be an essential behavior for the den master to attract females, but it has not been reported in Japanese or American giant salamanders.

### 4.2. Showering Behavior

We found that showering mainly occurred between 21:30–22:30, which was about 30 min later than the time (21:00–22:00) reported by Liang et al. (2010) [16], and is slightly different to the time (21:40–01:20) reported by Xu et al. (2013) [17]. This is possibly due to regional, seasonal or observation method differences. Five types of showering behavior involving the head, trunk, tail, curve, and snout were observed, which is similar to the report by Xu et al. (2013) [17], except for adherence showering. There was a significant positive correlation between showering and courtship behavior, due to showering promoting the development of the testis in males, triggering courtship behavior [17]. Water flow plays an important role in promoting the gonadal maturation of fish, which stimulates its sense organs, as is the case of *Acipenser schrenckii* Brandt and salamanders [20,21]. Snout showering raises the head, which may stimulate the hypothalamus and pituitary glands to secrete a gonadotropin-releasing hormone (GnRH) and gonadotropin, which can promote the release of sex hormones from the gonads [20,21]. These hormones promote the development of sexual cells and the occurrence of courtship behavior. Showering of the trunk, tail and curve directly stimulate the genital gland through a variety of gestures [20,21]. Therefore, water flow must be provided in their wild habitat or their ecological breeding stream during the breeding season to promote the male’s testis development through showering. Finding the appropriate water velocity requires further study.

### 4.3. Courtship

The courtship behavior of salamanders and newts can be divided into four stages: closing, chasing, following, and oviposition [21]. We found that knocking bellies, leaning side-by-side, riding, mouth-to-mouth posturing, chasing, rolling over, inviting, and cohabitation, were similar to the first three stages. Wild *A. davidianus* exhibited reproductive behaviors like biting and other activities before laying eggs. Pairs of *A. davidianus* swing and bite together with their mouths [13,14]. Liang et al. (2010) reported that courtship of *A. davidianus* included eight kinds of behavior, such as gathering together, exposing the head, inspection, following, cloacal sniffing, mouth-to-mouth posturing, inviting, and riding [16]. In our observations, riding, mouth-to-mouth posturing, chasing, and inviting were confirmed. Leaning side-by-side and knocking bellies were similar to gathering together; however, cloacal sniffing and the other behaviors were not found. We are the first to report rolling over and cohabitation. The courtship behavior of *A. davidianus* stimulated the gonadal development of males and females through body contact and friction, and promoted the synchronous development of germ cells [16,22]. Furthermore, courtship behavior may help them find suitable breeding partners.

### 4.4. Oviposition

Zhang et al. (2006) reported that male and female *A. davidianus* postured mouth-to-mouth, knocked bellies, touched each other, and mated in ponds, after they were injected with a sex hormone. Finally, bead-like eggs were oviposited and fertilized [15]. Our study found that the male and female entwined their tails and bellies, rotated in the den, and discharged bead-like eggs without hormones, in a simulated ecology farm [16,17]. This environment is close to the natural habitat of wild *A. davidianus*, and provided most of the required ecological factors for reproduction, so that *A. davidianus* could express its inherent behavior [22]. Physical contact between males and females may be essential during the process of amphibian mating, as it enables the male to transmit pheromones from its skin glands to the female in order to stimulate sexual desire and achieve reproductive synchronization [23,24].

### 4.5. Parental care

This study confirmed previous descriptions that male *A. davidianus* conduct parental care to safeguard eggs [12,13,16]. *A. japonicus* carry out tail fanning, agitation, and egg eating behaviors during the parental care process [25]. We found that *A. davidianus* exhibit all of these behaviors as well as shaking, which was first reported in *Andrias.* Shaking is close to the rocking of *A. japonicus,* where the four limbs are grounded in the same position [25], but the trunk of *A. davidianus* shakes up and down or right and left, which prevents eggs from being infected by water mold or the yolk from sticking to the shell, as well as coordinating other behavior [25]. We also found that tail fanning was the dominant behavior involved in the parental process, which increases the water flow and the content of dissolved oxygen (DO) in the water to satisfy the requirement of high DO for embryonic development [26]. It also prevents the yolk from sticking to the shell. Parental care behaviors during the middle stage were the most frequent (Figure 7), because the embryo was undergoing organogenesis [26], and needed more DO for a strong metabolism. Therefore, in order to satisfy the requirement of sufficient DO for embryo growth, the proper water speed and water quality should be ensured during the period of the egg hatching for artificial *A. davidianus* or its wild populations.

Our study found that the egg-eating behavior of *A. davidianus* differs from that of many fish, which is called parent-offspring cannibalism behavior [27]. *A. davidianus* mainly eats unfertilized, yolk-stuck and water-mold-infected eggs in order to reduce the potential contamination to neighboring eggs [25]. Egg eating during the late stage was more frequent than in the early/middle stage (*p* < 0.01), because there was an increased occurrence of unfertilized and impaired eggs and yolk sticking to the shell at that time (Figure 7). Thus, while *A. davidianus’* eggs were incubated artificially, the eggs needed to be turned over regularly, and those eggs that were unfertilized, had yolk sticking to the shell or exhibited saprolegniasis should be removed.

## 5. Conclusions

This study observed the reproductive behavior of *A. davidianus* using a digital monitoring system for the first time. It revealed the composition and characteristics of the reproductive behavior of *A. davidianus* and systematically categorized the types and time distribution of parental care, which can provide a scientific basis for the optimization of breeding methodologies in the ecological farming of *A. davidianus,* and the conservation of wild populations. This information also effectively promotes the progress of research into amphibian behavior and provides a reference for the quantitative study of animal behavior. In addition, it demonstrates that an infrared surveillance system is an effective research apparatus for studying the behavior of Cryptobranchidae and other cave animals. However, we only observed two pairs of *A. davidianus* successfully laying eggs and conducting parental care. A larger sample size and improved monitoring technique are essential for the further study of the reproductive behavior of *A. davidianus*. In addition, the behavior of *A. davidianus* should be observed in their wild habitat despite the inherent difficulties. In this way, their reproductive behavior may be better understood.

## Figures and Tables

**Table 1:** Description of the sand-pushing behavior of male *Andrias davidianus*.

Part of Body	Description of Posture	Duration of Each Continuous Gravel-Pushing (s)	Total Duration of Actions (h) and Coverage (%)
**Mouth**	Move gravel away from den with mouth (Figure 2a)	36.8 ± 31.8	4.2 (6.2%)
**Limbs**	Push gravel backward with fore or hind limbs, generally with head inside the den (Figure 2b)	58.79 ± 42.83	36.3 (53.8%)
**Trunk**	Scrape gravel away from body flanks with trunk (Figure 2c)	93.0 ± 71.2	2.1 (3.1%)
**Tail**	Shuffle silt or sand from the bottom of the den with tail (Figure 2d)	23.5 ± 12.4	24.9 (36.9%)

**Table 2:** Description of showering behavior in male *A. davidianus.*

Showering Behavior	Description	Frequency	Duration (min)/Percentage (%)
Head	Head rinsed with a swinging motion (Figure 3a)	8	3.52 ± 0.44/27.5
Trunk	All parts of the trunk rinsed with a swinging motion (Figure 3b)	7	4.23 ± 0.52/28.9
Tail	Tail rinsed with a swinging motion (Figure 3c)	6	4.02 ± 0.35/23.6
Curve	Body washed by bending around the water from the outlet (Figure 3d)	5	2.86 ± 0.25/14.0
Snout	Snout washed by moving the head out of the water (Figure 3e)	4	1.55 ± 0.16/6.1

**Table 3:** Description of courtship behavior of *A. davidianus*.

Courtship Behavior	Description	Frequency Percentage (%)	Duration Percentage (%)	Duration of Each Continuous Behavior (min)
**Side-by-side**	A male and female stand side-by-side, with heads touching.	21.58 ± 0.46	2.83 ± 0.46	2.49 ± 0.41
**Knocking bellies**	A male knocks the belly of his female partner with his snout and his head.	19.49 ± 0.30	2.61 ± 0.58	2.53 ± 0.48
**Riding**	A male climbs onto a female’s trunk. or head and places his head on the back of the female.	15.26 ± 0.37	2.41 ± 0.71	2.92 ± 0.51
**Mouth-to-mouth posturing**	A male and female touch each other with their snouts.	12.93 ± 1.03	2.06 ± 0.65	1.60 ± 0.24
**Chasing**	A male chases a female in the den.	10.78 ± 2.11	1.56 ± 0.37	3.04 ± 0.57
**Cohabiting**	A male and female live in the same den.	10.76 ± 0.58	88.32 ± 3.23	181.25 ± 38.67
**Inviting**	A male swims in and out of the den repeatedly until a female enters.	7.76 ± 0.68	1.77 ± 0.38	2.80 ± 0.43
**Rolling over**	A male turns his body over to show himself to a female.	1.41 ± 0.15	0.40 ± 0.14	4.71 ± 0.81

**Table 4:** Description of parental care in male *A. davidianus*.

Parental Care	Description	Duration (min/time)
**Tail fanning**	Tail swayed within or beside a pile of eggs like a fan (Figure 6a)	3.5 ± 0.6
**Agitating**	Head or body moved within a cluster of eggs (Figure 6b)	3.1 ± 0.6
**Egg eating**	The male ate the eggs that were unfertilized, had yolk stuck to shell or had water mold infection. This often followed agitating behavior (Figure 6c)	2.6 ± 0.8
**Shaking**	The trunk was placed above a pile of eggs, and shaken up and down or right and left with four limbs grounded on the same position (Figure 6d)	2.1 ± 0.3

## Appendix A

**Table A1:** Description of characteristics of *Andrias davidianus* used in research.

Sample Number		Total Length (m)	SVL	Weight (kg)	Age	Mating Success
**Partner A**	Male A	1.08	0.68	8.41	9	Yes
	Female A	1.02	0.64	8.23	9	Yes
**Partner B**	Male B	1.01	0.63	7.81	8	Yes
	Female B	1.06	0.66	8.34	9	Yes
**Partner C**	Male C	0.96	0.60	7.78	8	No
	Female C	1.05	0.66	8.25	9	No
**Partner D**	Male D	0.91	0.57	5.86	8	No
	Female D	0.95	0.60	6.37	8	No
